# Multi-feature computational framework for combined signatures of dementia in underrepresented settings

**DOI:** 10.1088/1741-2552/ac87d0

**Published:** 2022-08-25

**Authors:** Sebastian Moguilner, Agustina Birba, Sol Fittipaldi, Cecilia Gonzalez-Campo, Enzo Tagliazucchi, Pablo Reyes, Diana Matallana, Mario A Parra, Andrea Slachevsky, Gonzalo Farías, Josefina Cruzat, Adolfo García, Harris A Eyre, Renaud La Joie, Gil Rabinovici, Robert Whelan, Agustín Ibáñez

**Affiliations:** 1 Global Brain Health Institute (GBHI), University of California San Francisco (UCSF), CA, United States of America; 2 Cognitive Neuroscience Center (CNC), Universidad de San Andrés, Buenos Aires, Argentina; 3 Latin American Brain Health (BrainLat), Universidad Adolfo Ibáñez, Santiago, Chile; 4 National Scientific and Technical Research Council (CONICET), Buenos Aires, Argentina; 5 Department of Physics, University of Buenos Aires, Buenos Aires, Argentina; 6 Medical School, Aging Institute, Psychiatry and Mental Health, Pontificia Universidad Javeriana, Bogota, Colombia; 7 MAP: School of Psychological Sciences and Health, University of Strathclyde, Glasgow, United Kingdom; 8 Gerosciences Center for Brain Health and Metabolism, Santiago, Chile; 9 Faculty of Medicine, University of Chile, Santiago, Chile; 10 Memory and Neuropsychiatric Clinic (CMYN) Neurology Department, Hospital del Salvador and University of Chile, Santiago, Chile; 11 Servicio de Neurología, Departamento de Medicina, Clínica Alemana-Universidad del Desarrollo, Santiago de Chile, Chile; 12 Departamento de Lingüística y Literatura, Facultad de Humanidades, Universidad de Santiago de Chile, Santiago, Chile; 13 Neuroscience-Inspired Policy Initiative, Organisation for Economic Co-operation and Development and PRODEO Institute, Paris, France; 14 IMPACT, The Institute for Mental and Physical Health and Clinical Translation, Deakin University, Geelong, Victoria, Australia; 15 Department of Psychiatry and Behavioral Sciences, Baylor College of Medicine, Houston, TX, United States of America; 16 Memory and Aging Center, Department of Neurology, Weill Institute for Neurosciences, University of California, San Francisco, San Francisco, CA, United States of America; 17 Trinity College Dublin, Dublin, Ireland

**Keywords:** multimodal neuroimaging, neurodegeneration, harmonization, feature selection, machine learning

## Abstract

**Objective.:**

The differential diagnosis of behavioral variant frontotemporal dementia (bvFTD) and Alzheimer’s disease (AD) remains challenging in underrepresented, underdiagnosed groups, including Latinos, as advanced biomarkers are rarely available. Recent guidelines for the study of dementia highlight the critical role of biomarkers. Thus, novel cost-effective complementary approaches are required in clinical settings.

**Approach.:**

We developed a novel framework based on a gradient boosting machine learning classifier, tuned by Bayesian optimization, on a multi-feature multimodal approach (combining demographic, neuropsychological, magnetic resonance imaging (MRI), and electroencephalography/functional MRI connectivity data) to characterize neurodegeneration using site harmonization and sequential feature selection. We assessed 54 bvFTD and 76 AD patients and 152 healthy controls (HCs) from a Latin American consortium (ReDLat).

**Main results.:**

The multimodal model yielded high area under the curve classification values (bvFTD patients vs HCs: 0.93 (±0.01); AD patients vs HCs: 0.95 (±0.01); bvFTD vs AD patients: 0.92 (±0.01)). The feature selection approach successfully filtered non-informative multimodal markers (from thousands to dozens).

**Results.:**

Proved robust against multimodal heterogeneity, sociodemographic variability, and missing data.

**Significance.:**

The model accurately identified dementia subtypes using measures readily available in underrepresented settings, with a similar performance than advanced biomarkers. This approach, if confirmed and replicated, may potentially complement clinical assessments in developing countries.

## 1. Introduction

Global approaches to dementia should address the diversity and heterogeneity of poorly characterized, underdiagnosed populations, including Latinos. Despite having greater dementia risk, ethnoracially diverse groups are systematically underrepresented in research and clinical trials [1]. Dementia prevalence is notably high among diverse populations from upper middle-income countries and LMICs, including SACs [2]. Relative to US and European samples, SACs present more heterogeneous populations with shorter lifespans, a preponderance of non-urban backgrounds, lower education level and SES [3], and, crucially, greater variability in dementia presentation [2]. Considering the impact of genetic, SES [3], and environmental risk factors in phenotypic heterogeneity across ethnic groups from SACs, multidimensional studies are urgently needed in the region [3, 4].

Mainstream dementia frameworks rely on underlying pathological biomarkers such as *β*-Amyloid and tau PET neuroimaging [5]. However, budgetary and access constraints limit the use of biomarker approaches in SACs [2, 6]. Moreover, as dementia presentation may depend on multiple factors (such as genetics [7], SES [3, 4], and environmental risk factors [8]), unimodal characterization by a single biomarker may then prove ineffective. Multimodal markers may help to bridge this gap particularly in SACs because neurodegenerative diseases usually present heterogeneous profiles across different levels [9]. In particular, the combination of multifactorial sources of variability [3] may induce atypical presentations of AD and bvFTD. In order to capture the broad spectrum of dementia presentation and heterogeneity, cognitive assessments [10], structural MRI [11], EEG [12], and rs-fMRI [13] markers are widely available across countries in the world, even in underrepresented populations [9, 10]. Unlike traditional univariate approaches, machine learning facilitates the modeling of complex interactions between variables across heterogeneous datasets [14]. Automatized diagnostic methods used as decision support tools have shown promising results in dementia [15], especially with high dimensionality methods that proven superior than classical statistical models [16]. Therefore, an automatized machine-learning approach to multimodal markers can potentially overcome current limitations in the characterization of populations from SACs and other underrepresented regions.

**Figure 1.**
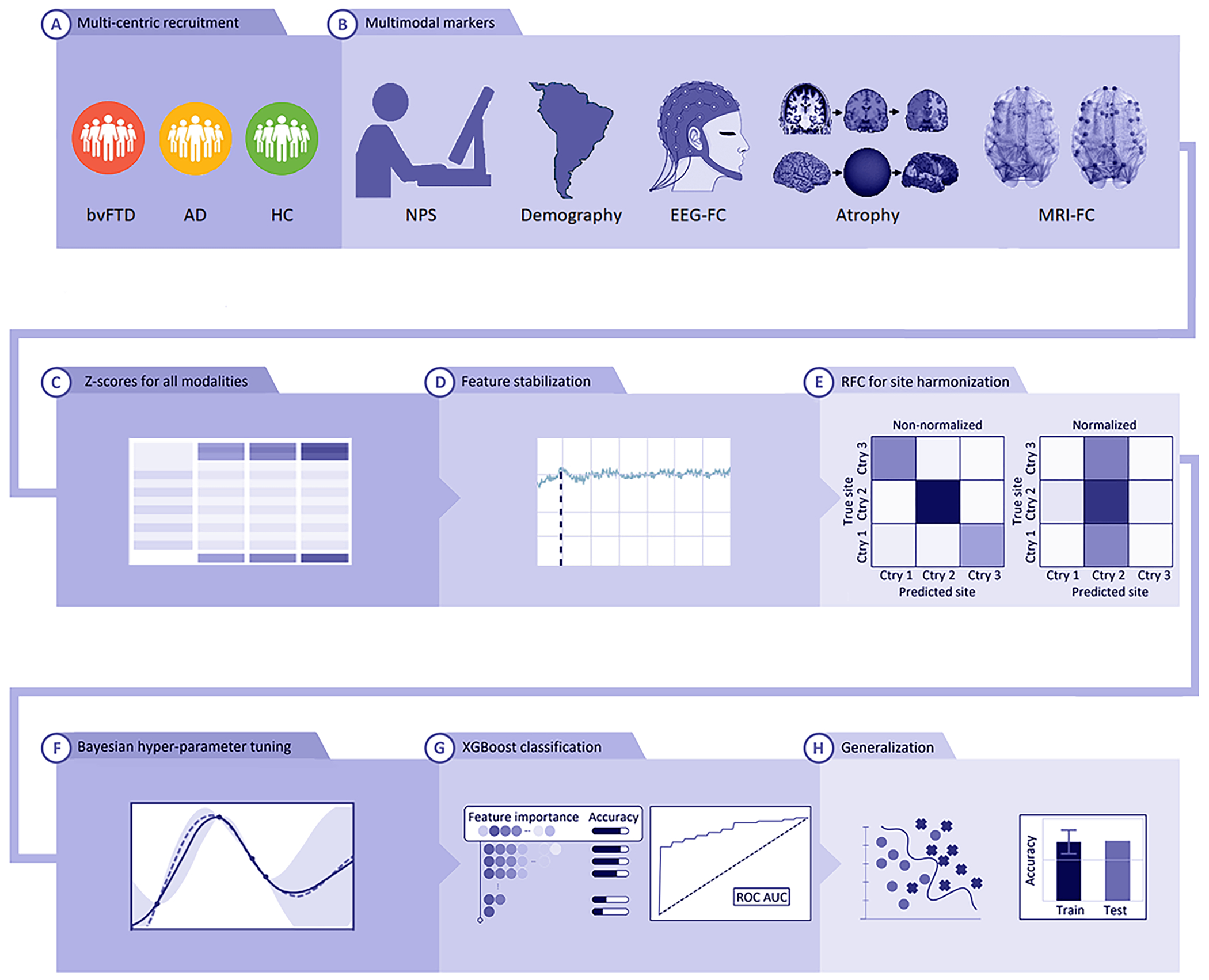
MMAN (A) recruitment from three centers consisted of of 54 bvFTD patients, 76 AD patients, and 152 HCs. (B) Acquisition of multimodal markers consisting of NPSs, DEMs (sex, age, years of education), EEG-FC, atrophy markers, and MRI-FC markers. (C) Normalization of all modalities via *z*-scores. (D) Feature stabilization techniques using recursive feature elimination to find the optimal set of features. (E) RFC approach to test for unbiased results by classifying relative to the images’ site of origin. (F) For testing different feature combinations, we used a *k*-fold (*k* = 10) validation scheme for Bayesian hyper-parameter tuning to obtain trained XGBoost models. (G) For receiver operating characteristic (ROC) analysis, we defined bvFTD group as the ‘positive’ class and AD group as the ‘negative’ class, allowing the sensitivity and specificity metrics being applicable to patient group comparisons, and feature importance analysis results. (H) Generalization results using an out-of-sample set. BvFTD: behavioral-variant frontotemporal dementia; AD: Alzheimer’s disease; HCs: healthy controls; NPS: neuropsychological cognitive and executive markers; EEG-FC: EEG functional connectivity; MRI-FC: MRI functional connectivity; RFC: random forest classifiers.

Here, we developed a multi-feature multimodal approach to neurodegeneration (MMAN, figure 1) from diverse samples of AD, bvFTD, and controls from SACs. We combined demographic (DEM) information, neuropsychological outcomes (NPSs) (cognitive screening, executive functions), structural MRI atrophy measures, and dynamic functional connectivity (FC) metrics from EEG and fMRI in an integrative approach using the eXtreme Gradient Boosting (XGBoost) machine learning classifier [17, 18]. The XGBoost parameters were tuned by Bayesian optimization, including a data harmonization technique to remove possible site-specific biases [19]. The differential characterization of dementia subtypes such as AD vs FTD presents several challenges (cf controls vs patients [17]), including overlapping atrophy patterns and variability in cognitive and neuroimaging measures among dementia subtypes [20, 21]. To overcome these limitations, we combined cognitive screening, MRI-based morphometry, measures of EEG connectivity [22], and dynamic measures of resting-state fMRI connectivity [23]. To this end, we assessed the relative weights of each feature (i.e. different markers of cognitive screening, EEG, MRI, ad fMRI) for a combined classification of AD and bvFTD using a technique from machine learning called feature importance analysis. Most of multimodal machine learning approaches for AD characterization [24–28] focusing on MRI data come from high-income countries such as the Alzheimer’s Disease Neuroimaging Initiative (ADNI) [29] and Open Access Series of Imaging Studies (OASIS) [30] databases, where both neuroimaging parameters and sample DEMs are homogeneous. Conversely, our work is developed for real-life clinical scenarios with heterogenous acquisition parameters and patients’ diversity across SACs. To the best of our knowledge, this is the first multimodal approach intended for a differential characterization between two dementia subtypes.

We implemented a mixed hypothesis- and data-driven approach, including *a priori* predictions based on the literature and machine learning analyses of relevant multimodal data. First, we hypothesized that the XGBoost classifier would yield high accuracy to classify patients vs controls, but also AD vs FTD patients considering clinically relevant multimodal features. Second, we anticipated that the most important features to characterize dementia would have maximal predictive power with combined multimodal measures at different levels (cognitive, atrophy, EEG/fMRI connectivity). Third, we predicted that the MMAN would outperform all unimodal approaches in classifying patients from controls and AD from FTD patients. Moreover, classification performance would remain high even when considering (a) DEMs (sex, age, years of education) as a source of variability, (b) strong reduction in the number of features (from thousands to dozens), (c) missing data, and (d) multimodal sources of variability across clinical centers. By testing these hypotheses we aim to assess the robustness of a multimodal computational framework for characterizing neurodegenerative diseases in underrepresented populations.

## 2. Methods

### 2.1. Participants

This study comprised 282 participants from a multicenter protocol [4, 9] with sites in Argentina (Country-1), Chile (Country-2), and Colombia (Country-3). All centers used the standardized diagnostic assessment of the Multi-Partner Consortium to Expand Dementia Research in Latin America (ReDLat) [4, 9]. Clinical diagnoses were established by experts in dementia through an extensive neurological and neuropsychiatric examination comprising semi-structured interviews and standardized assessments, with current criteria for probable bvFTD [31], and NINCDS-ADRDA clinical criteria for AD [32]. We also included 152 HCs, matched on age, sex, and education with the patient groups (table 1). However, given subtle DEM differences, age and education were also included in the machine learning pipeline. All participants provided written informed consent following the Declaration of Helsinki. Each institutional Ethics Committee approved the protocol.

In clinical settings, and specially across SAC’s sites, patient’s incomplete evaluations and assessment commonly occurs. Thus, we evaluated whether our MMAN model was robust against missing data in a fraction of subjects and features. As some centers may not have access to specific assessments, missing information can constitute an obstacle for this approach. We tested the same pipeline on a sub-sample (SS) without missing data and on a full-sample (FS) with missing data. The SS consisted of 54 HCs (18 from Country-1, 20 from Country-2, and 16 from Country-3), 19 patients with bvFTD (7 from Country-1, 7 from Country-2, and 5 from Country-3), and 32 patients with AD (9 from Country-1, 10 from Country-2, and 13 from Country-3). The FS consisted of 152 HCs (51 from Country-1, 49 from Country-2, and 52 from Country-3), 54 patients with bvFTD (16 from Country-1, 20 from Country-2, and 18 from Country-3), and 76 patients with AD (25 from Country-1, 24 from Country-2, and 27 from Country-3). The FS was not completely balanced in DEM data, allowing us to test whether the classifier is robust even in the presence of these unmatched variables. To handle missing data in the FS, we used feature averaging imputation on the features that contained up to 30% missing values [33] (table 2). To this end, we used a single averaged value per feature to be imputed in the table fields of the subjects having missing values.

### 2.2. Cognitive markers (cognitive screening and executive functions)

The Montreal Cognitive Assessment (MoCA) [34] is a brief cognitive screening instrument that evaluates attention and concentration, abstraction, object recognition, executive functions, memory, language, visuoconstructional and visuospatial skills, conceptual thinking, calculations, and orientation (maximum score = 30, higher scores indicate better performance). The MoCA can track cognitive decline in patients with neurodegenerative diseases. The INECO Frontal Screening (IFS) [35] is a 10 min, easy-to-administer executive functions screening tool. It includes eight subtests, assaying three executive functions: response inhibition and set shifting (four tasks), working memory (three tasks), and abstraction capacity (one task). The maximum score is 30, higher scores indicate better performance. The IFS is sensitive and specific for detection frontal-executive dysfunction in patients with neurodegenerative diseases [35]. The MoCA and the IFS were not considered for the patient’s diagnostic procedures.

### 2.3. EEG markers

Participants completed a 10 min long high density EEG acquisition, on a 128-channel system with preamplified sensors and a DC coupling amplifier, at a sampling rate of 1024. Across centers, data were recorded via Biosemi Active-two 128-channel systems with pre-amplified sensors and a DC coupling amplifier, at a sampling rate of 1024 Hz. Analog filters were set at 0.03 and 100 Hz. A digital bandpass filter between 0.5 and 45 Hz was applied offline to remove unwanted frequency components. The reference was set to link mastoids for recordings and re-referenced offline to the average of all electrodes. Eye movements or blink artifacts were corrected with independent component analysis [36] and with a visual inspection protocol [37–39]. Bad channels were replaced via statistically weighted spherical interpolation (based on all sensors) [40]. The data was divided in 1000 ms segments from the beginning until the end of the recording. All EEG signal processing steps were implemented on MATLAB software (vR2016a) through the EEGLAB (v14.1.2) [41] toolbox. During the 10 min long resting state protocol, participants were instructed not to think about anything in particular while keeping awake, still, and with eyes closed. We measured linear interactions between oscillatory signals using phase-locking value (PLV) [22] and non-linear information sharing via the weighted symbolic mutual information (wSMI) metric [42]. Connectivity was averaged across segments to create the adjacency matrix. To reduce the number of features while preserving topographic specificity, we defined 16 regions of interest (ROIs) of eight electrodes for each lobe and hemisphere. To quantify the strength of between- and within-ROI connections, we estimate the averaged connectivity values of all inter-electrode connections linking electrodes in any two ROIs or within a ROI, respectively.

### 2.4. Structural and functional MRI markers

We obtained three-dimensional structural volumetric and 10 min long resting state MRI sequences from all participants—recordings were performed in three scanners (table 3). MRI cortical thickness metrics and volumetric estimates included voxel-based and surface-based morphometry (SBM) [11]. The structural volumetric analysis preprocessing included removal of non-brain tissue, an automatic Talairach transformation, segmentation of the subcortical white matter (WM) and deep grey matter (GM) volumetric structures (including hippocampus, amygdala, caudate, putamen, and ventricles), intensity normalization, tessellation of the GM-WM boundary, an automatic topology correction, and surface deformation following intensity gradients to optimally place the GM/WM and GM/cerebrospinal fluid (CSF) borders at the location where the greatest shift in intensity defines the transition to the other tissue class. All T1 images were processed via SBM on FreeSurfer software suite (v 6.0, https://surfer.nmr.mgh.harvard.edu/). Structural surface-based metrics included cortical volume and thickness. SBM avoids registration to a standard space, overcoming registration errors, improving parcellation, and offering reliable estimation of region-specific differences [43]. Once the cortical models were processed, additional procedures were performed for further analysis, including surface inflation, registration to a spherical atlas-based on individual cortical folding patterns—parcellation of the cerebral cortex into units relative to gyral and sulcal structure, and creation of a variety of surface-based data—including maps of curvature and sulcal depth. These methods use both intensity and continuity information of the entire 3D magnetic resonance (MR) volume from segmentation and deformation procedures to produce representations of cortical thickness, which is calculated as the closest distance from the GM/WM boundary to the GM/CSF boundary at each vertex on the tessellated surface. The maps were created using spatial intensity gradients across tissue classes; therefore, they were not simply reliant on absolute signal intensity. Since the ensuing maps were not restricted to the voxel resolution of the original data, they can detect submillimeter differences between groups. FreeSurfer’s morphometric procedures have been demonstrated to show good test–retest reliability across scanner manufacturers and field strengths. Full details on the implemented methods can be found elsewhere [44]. Finally, the volume, area, and thickness from each segmentation based on the Desikan–Killiany parcellation of cortical and subcortical areas [45] were quantified. The plain-text output of the FreeSurfer’s pipeline was post-processed on Python (version 3.7.4, Python Software Foundation) and transformed into a better structure for statistical analysis. To avoid potential biases due to differences among the participants’ head size [46], volume measures of each area were normalized as a percentage of the estimated total intracranial volume (provided also in FreeSurfer’s results).

For the resting-state protocol, participants were asked not to think about anything in particular, to keep their eyes closed, and to avoid moving or falling asleep. In each center, we obtained three-dimensional volumetric and 10 min long resting-state MRI sequences from all participants. First, to ensure that magnetization achieved a steady state, we discarded the first five volumes of each subject’s resting-state recording. Then, images were pre-processed in MATLAB using an open-access toolbox: the Data Processing Assistant for Resting-State fMRI (DPARSF V2.3) [47], which generates an automatic pipeline for fMRI analysis by calling the Statistical Parametric Mapping software (SPM12) [48] and the Resting-State fMRI Data Analysis Toolkit (REST V. 1.7 toolbox) [49]. The images were slicetime corrected (using as reference the middle slice of each volume) and aligned to the first scan of the session to correct head movement. To reduce the effects of motion and physiological artifacts, six head-motion parameters, as well as WM and CSF signals, were removed as nuisance variables. WM and CSF masks for this procedure were derived from the tissue segmentation of each subject’s T1 scan in native space. As an additional analysis, we calculated the frame-wise displacement (FD) [50] to check for head movement differences between groups. This measure, that indexes the movement of the head from one volume to the next, is calculated as the sum of the absolute values of the differentiated realignment estimates (by backwards differences) at every timepoint. After calculating FD for each group, no statistically significant differences were found after an ANOVA test (table 4). Using the pre-processed rs-fMRI time series as input, we captured static and linear associations using Pearson’s R static FC (SFC) [51]. We also performed a non-linear dynamic connectivity fluctuation analysis (DCFA) [23]. This method captures dynamic FC fluctuations [52], allowing for time-dependent connectivity analysis instead of averaging connectivity across the whole recording. We focused our analyses on five well-known and standard resting-state networks. The default mode network (DMN), the salience network (SN), the executive network (EN), the visual network, and the motor network [53].

### 2.5. Machine-learning methods

To limit biases and obtain more representative results, we employed a *k*-fold validation approach (*k* = 10) using 80% of the sample for training and validation, and 20% out-of-folds sample as an independent test-set. This testing dataset was never used for hyperparameter tuning, data reduction or feature engineering to evaluate the generalizability of our results. First, we performed a site normalization process for each feature of both HCs and patients via *z*-scores based on the mean and standard deviation of the corresponding center’s HCs. This process was applied within each fold to avoid information leakage (figure 1(C)). Afterwards, we performed feature stabilization by forward sequential feature selection [54] to obtain the best subset of features for each subject-group classification pair (figure 1(D)). For this, we optimized the accuracy of a random forest classifier (RFC) varying the number of features sequentially from a single one to all features according to its classificatory relevance. This classifier quantifies the importance of a feature depending on how much the average Gini impurity index decreases in the forest due to its use as node in a tree. This process was employed for both the FS (1523 features, while imputing the average on missing data), and the SS (1513 features without missing data). On each step for feature sets evaluation, we employed a RFC on default hyper-parameters [54] to evaluate classification accuracy based on a *k*-fold cross validation (*k* = 10). We used the Gini scores to eliminate features by removing features with the lowest importance at each iteration and checked for the robustness of our results based on the final number of features after stabilization for both samples. Finally, we kept the *N* first features in the ranking, where *N* is the optimal number of features such that using more than *N* features fails to improve classifier’s performance. Afterwards, to evaluate if the results were unbiased with respect to the acquisition site, we performed an RFC analysis (on default hyperparameters) to check if the confusion matrices were yielding non-significant results (figure 1(E)).

Finally, we used the XGBoost [17] classifier, tuned by Bayesian hyper-parameter optimization (figure 1(G)), to obtain the patient group classification. The XGBoost algorithm is a gradient boosting machines (GBMs) implementation that provides parallel computation tree boosting, enabling fast and accurate predictions which have proven successful in several fields [55–57]. GBMs are based on the gradient boosting technique, in which ensembles of decision trees iteratively attempt to correct the classification errors of their predecessors by minimizing a loss function (i.e. a function representing the difference between the estimated and true values) pointing in the negative gradient direction [58]. When compared to other GBM algorithms, XGBoost provides regularized boosting, helping to reduce overfitting and thus providing more generalizable results [57, 59]. For a fast and accurate machine learning model hyper-parameter tuning on big datasets comprising of several features, we employed Bayesian optimization [60, 61]. The XGBoost has several hyper-parameters, such as the learning rate, the minimum loss reduction required to make a further partition of a leaf node, the maximum depth of a tree, the maximum number of leaves, and the regularization weights. In order to choose the best parameters for the classification in this high dimensional hyper-parameter space, we used Bayesian optimization [60, 61] (figure 1(F)). This state-of-the-art optimization framework demonstrated wide applicability to different problem settings. This is an iterative algorithm with two key ingredients: a probabilistic surrogate model and an acquisition function to decide which point to evaluate next. At each step, a new point of the hyper-parameter space to explore is selected to be the maximum of an activation function of the prior knowledge and the uncertainty. As this optimization progresses, the chances of finding a better solution increase. Compared to other techniques such as the grid-search which is undermined by issues of dimensionality or random-search (where each guess is independent from the previous run), the Bayesian optimization algorithm is fast to compute, enabling a thorough optimization of the hyper-parameters. To evaluate our classification results, we used the area under the curve (AUC) of the ROC curve. The confidence intervals were obtained with bootstrapping by resampling 5000 times [54].

## 3. Results

### 3.1. Feature optimization and harmonization results for the SS

**Figure 2.**
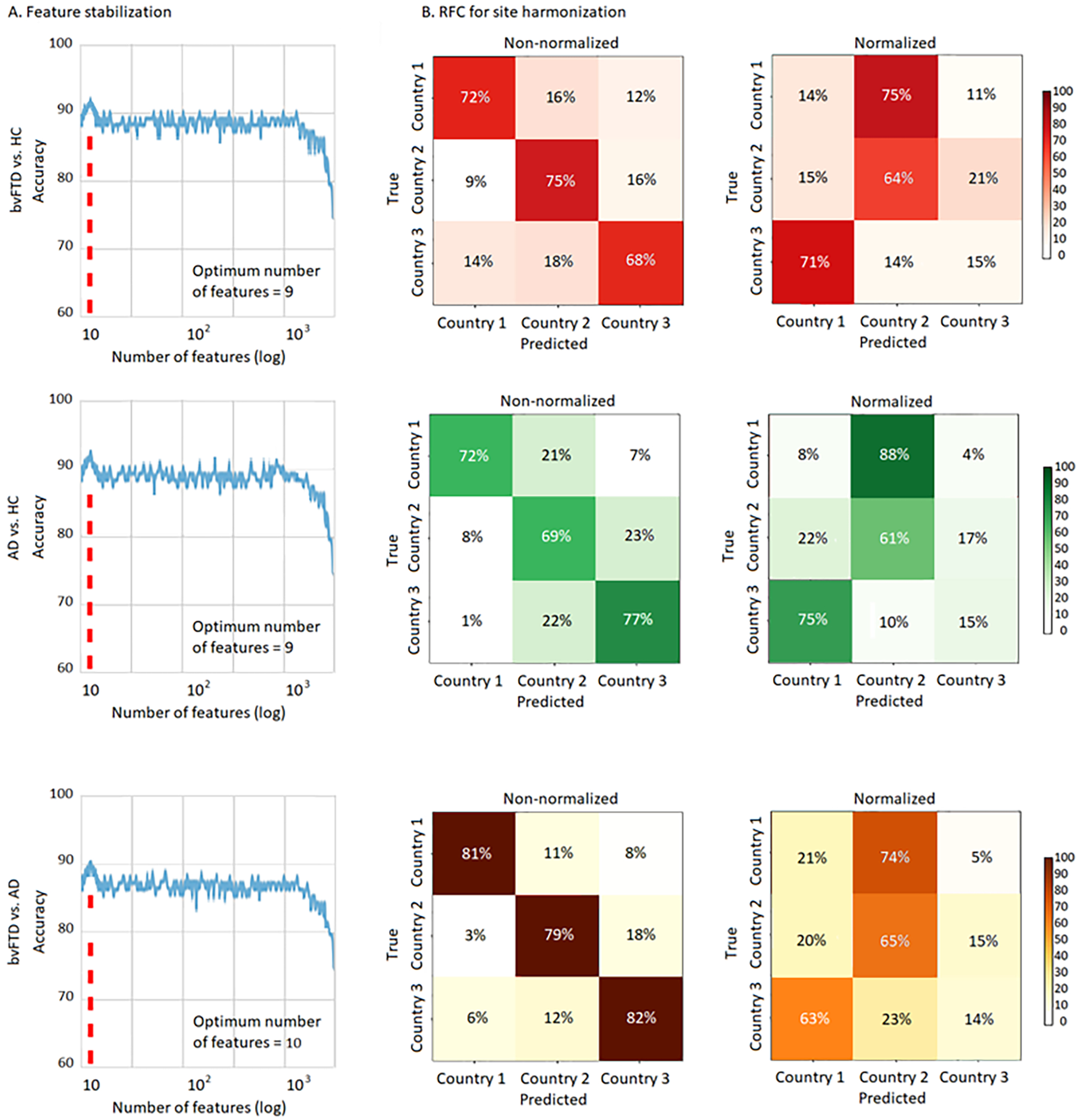
Feature stabilization and RFC analysis in the SS. (A) Feature stabilization curve for classification between bvFTD patients and HCs, AD patients and HCs, and bvFTD and AD patients, showing accuracy vs number of features in a logarithmic scale. Starting with a set containing all features available in the SS and finally keeping the set of features yielding maximal accuracy The optimal number of features for each classification pair and sample is highlighted in a discontinuous red line. (B) RFC analysis results for the non-normalized and normalized samples. A high accuracy rate was observed for classifying subjects per acquisition site prior normalization, and a non-statistically significant classification result for the normalized samples, confirming unbiased results. BvFTD: behavioral variant frontotemporal dementia; AD: Alzheimer’s disease; HCs: healthy controls; Log: logarithmic scale.

First, we applied the progressive feature elimination technique for the bvFTD vs HCs classification in the SS without missing data. For this classification pair, we obtained an optimal number of nine specific features that gave a maximum mean validation accuracy of 91.6% (±1.5%) (figure 2(A), first row). The site-harmonization processing yielded non-statistically significant confusion matrices for each country-wise classification after normalization (*p* > 05) (figure 2(B), second column) confirming unbiased results. For the classification between AD patients and HCs, we also obtained an optimal number of nine features, yielding a maximum mean validation accuracy of 92.2% (±4.3%) (figure 2(A), second row). The harmonization analysis showed a non-statistically significant country classification at chance level (figure 2(B), second column). Finally, for the classification between bvFTD and AD patients, we obtained an optimal number of ten features after stabilization, with a maximum mean validation accuracy of 91.7% (±2.1%) (figure 2(A), third row). Here, too, the confusion matrix also revealed non-significant results for each country after normalization (figure 2(B), second column).

### 3.2. Patient group classification in the SS

**Figure 3.**
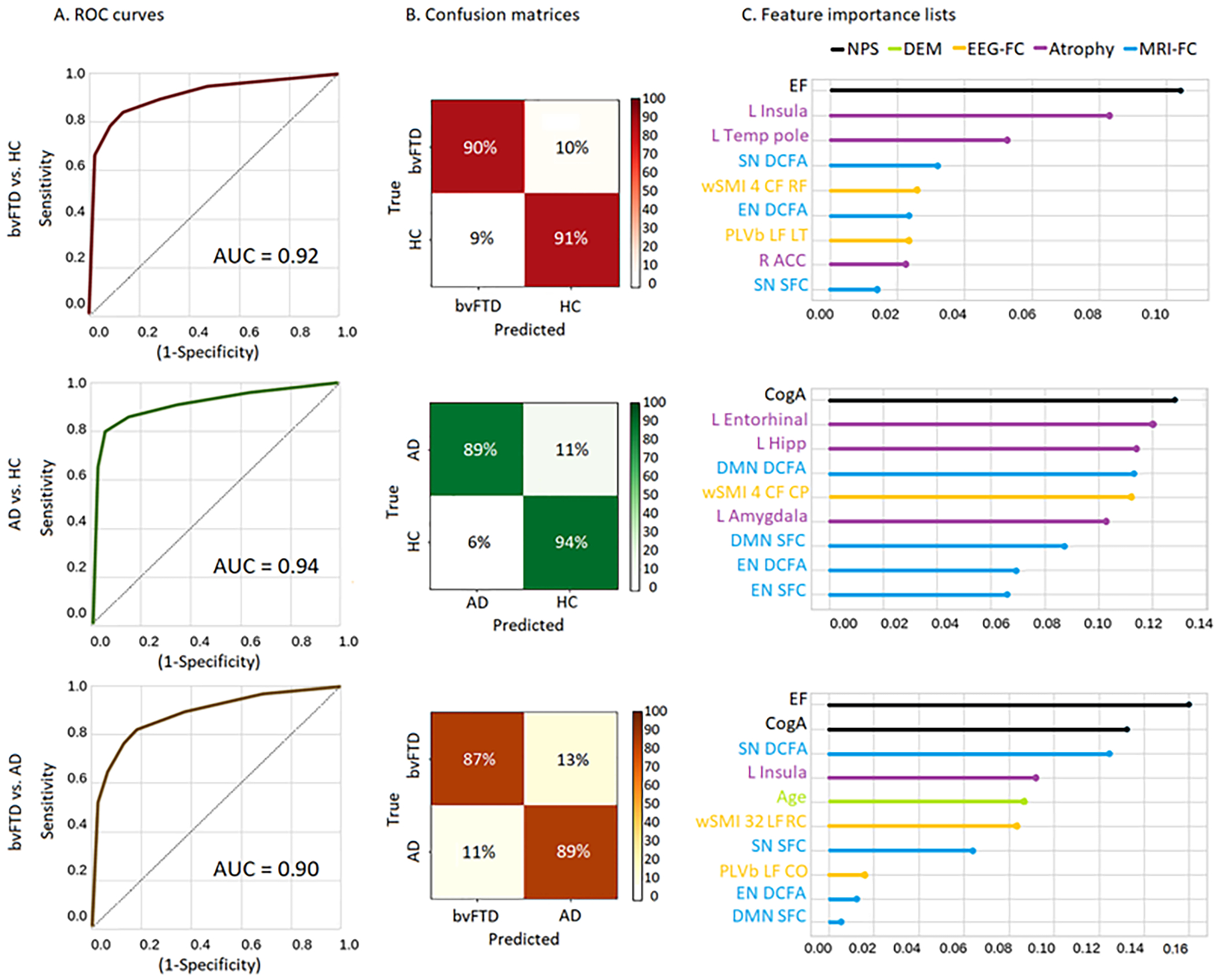
MMAN results for the SS. Machine learning results. (A) BvFTD patients vs HCs. ROC curve indicating specificity (true positive rate) and sensitivity (false positive rate), while calculating the AUC. Confusion matrix for true label and predicted label accuracy details. Feature importance plot of the most relevant features for the classification. Results show an AUC of 0.92, a sensitivity of 90%, and a specificity of 91%, with the EF total value as the top feature, followed L Insula and L Temp Pole as the top-three features. (B) AD patients vs HCs. ROC curve indicating specificity (true positive rate) and sensitivity (false positive rate), while calculating the AUC. Confusion matrix for true label and predicted label accuracy details. Feature importance plot of the most relevant features for the classification. Results yielded an AUC of 0.94, with a sensitivity of 89% and a specificity of 94%. The CogA total value resulted in the most important feature, followed by L Entorhinal and L Hipp as the top-three features (C) bvFTD vs AD patients. ROC curve indicating specificity (true positive rate) and sensitivity (false positive rate), while calculating the AUC. Confusion matrix for true label and predicted label accuracy details. Feature importance plot of the most relevant features for the classification. Results yielded an AUC of 0.90, a sensitivity of 87%, and a specificity of 89%, with the EF total value as the top feature, followed by CogA total and the SN DCFA as the top-three features. ROC: receiver operating characteristic; AUC: area under the curve; bvFTD: behavioral variant frontotemporal dementia; AD: Alzheimer’s disease; HCs: healthy controls; EF: executive functions; CogA: cognitive assessment; L Insula: left insula; L Temp Pole: left temporal pole; DCFA: dynamic functional connectivity analysis; SN: salience network; DMN: default mode network; EN: executive network; wSMI: weighted symbolic mutual information; PLVb: phase-locking value in the beta band; CF: central-frontal; LT: left-temporal; RF: right-frontal; L amygdala: left-amygdala.

After obtaining an optimized subset of features for each classification pair and checking that our results were unbiased site-wise, we tested the robustness of the machine learning classifier on the patient’s SS dataset. The machine learning classifier when applied on bvFTD patients and HCs yielded an AUC of 0.92 (±0.01) in the test set, with a sensitivity of 90% (±3%), and a specificity of 91% (±1%), In the feature importance list, the executive functions total score resulted as the top feature, followed by the left insula atrophy, left temporal pole atrophy, the nonlinear SN, the nonlinear wSMI EEG connectivity from the central-frontal to the right-frontal region, the nonlinear DCFA measure of the EN, the EEG PLV linear connectivity in the beta band from the left-frontal to the left-temporal region, the right anterior cingulate-cortex atrophy, and the linear SFC measure in the SN (figure 3, first row). For the classification between AD patients and HCs, we obtained an AUC of 0.94 (±0.01) in the test set, with a sensitivity of 89% (±2%) and a specificity of 94% (±1%). In the feature importance list, the cognitive assessment (total score) constituted the most important feature, followed by atrophy in the left entorhinal cortex, atrophy in the left hippocampus, the nonlinear DMN, the nonlinear EEG marker with from the centra-frontal to the left-frontal region, left amygdala atrophy, the linear DMN, the nonlinear EN, and finally, the linear EN (figure 3, second row). Lastly, for the classification between bvFTD and AD patients, the AUC was of 0.90 (±0.01) in the test set, with a sensitivity of 87% (±2%), and a specificity of 89% (±3%). The executive function total score was the top feature, followed by cognitive assessment (total score), the nonlinear SN, the left insular atrophy, age (DEM scores), non-linear EEG connectivity from the left-frontal to the right-central region, the linear SN, the linear EEG connectivity in the beta band from the left-temporal to the central-occipital region, the nonlinear EN, and the linear DMN (figure 3, third row).

### 3.3. Feature optimization and harmonization results for the FS

**Figure 4.**
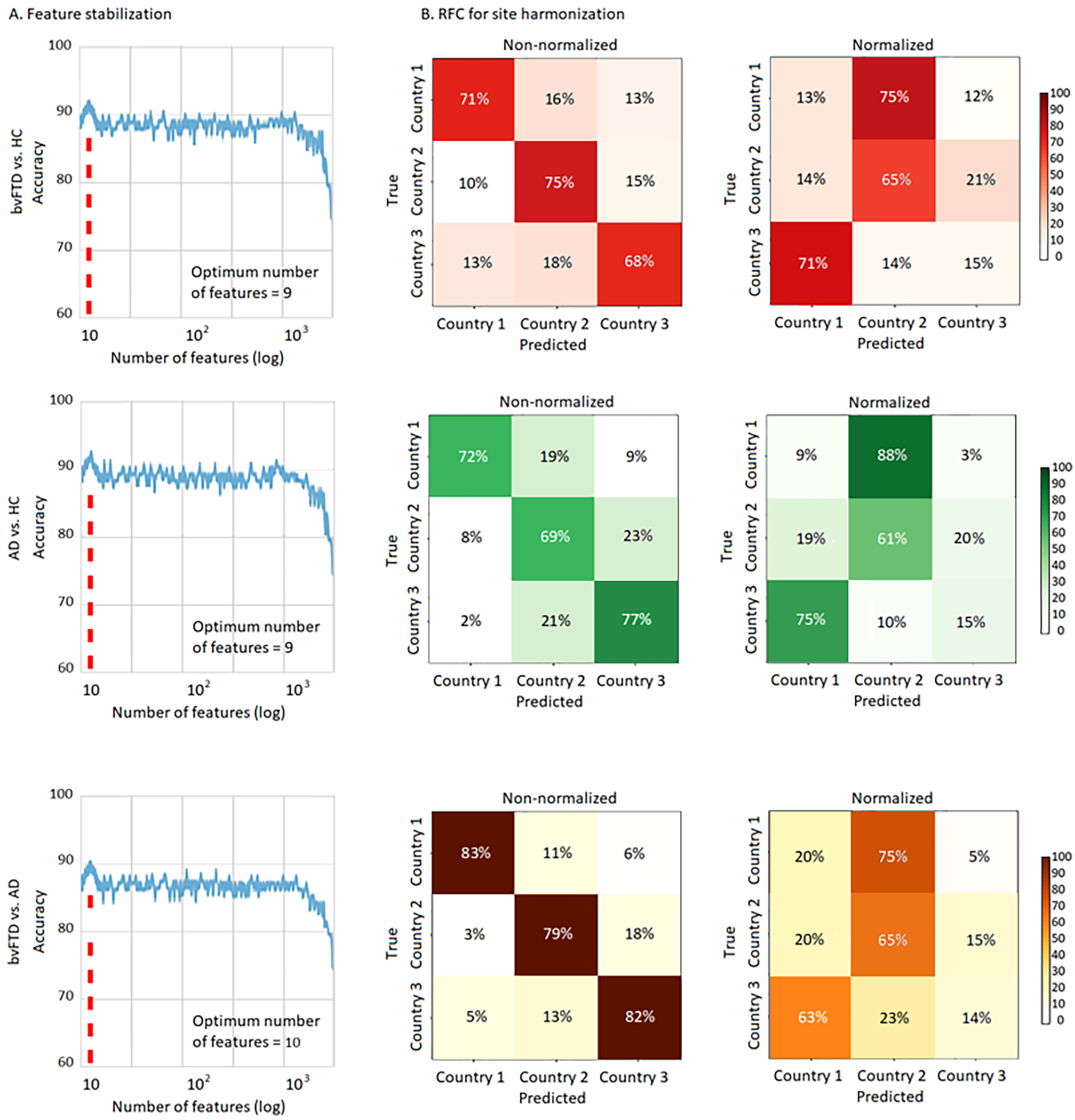
Feature stabilization and RFC analysis in the FS. (A) Feature stabilization curve for classification between bvFTD patients and HCs, AD patients and HCs, and bvFTD and AD patients, showing accuracy vs number of features in a logarithmic scale. Starting with a set containing all the features available in the FS and finally keeping the set of features yielding the best accuracy The optimal number of features for each classification pair and sample is highlighted in a discontinuous red line. (B) RFC analysis results for the non-normalized and normalized samples. High accuracy rates were obtained for classifying subjects per acquisition site prior normalization, and a non-significant classification result for the normalized samples, confirming unbiased results. BvFTD: behavioral variant frontotemporal dementia; AD: Alzheimer’s disease; HCs: healthy controls; Log: logarithmic scale.

For the machine learning classification between bvFTD patents and HCs in the FS dataset that had missing data, we obtained an optimal number of nine features after the optimization, resulting in a maximum mean validation accuracy of 91.1% (±2.3%) (figure 4(A), first row). The site-harmonization processing yielded a non-statistically significant confusion matrices for each country-wise classification after normalization (*p* > 05) (figure 4(B), second column), confirming unbiased results. For the classification between AD patients and HCs, we also obtained an optimal number of nine features, with a maximum mean validation accuracy of 92.3% (±1.6%) (figure 4(A), second row). The harmonization process also yielded a non-statistically significant country classification after normalization (figure 4(B), first column). Finally, for the classification between bvFTD and AD patients, we obtained an optimal number of ten features, with a maximum mean validation accuracy of 91.9% (±2.4%) (figure 4(A), third row). The harmonization analysis again showed a non-significant profile in the confusion matrix for each country after normalization (figure 4(B), second column).

### 3.4. Patient group classification with the FS

**Figure 5.**
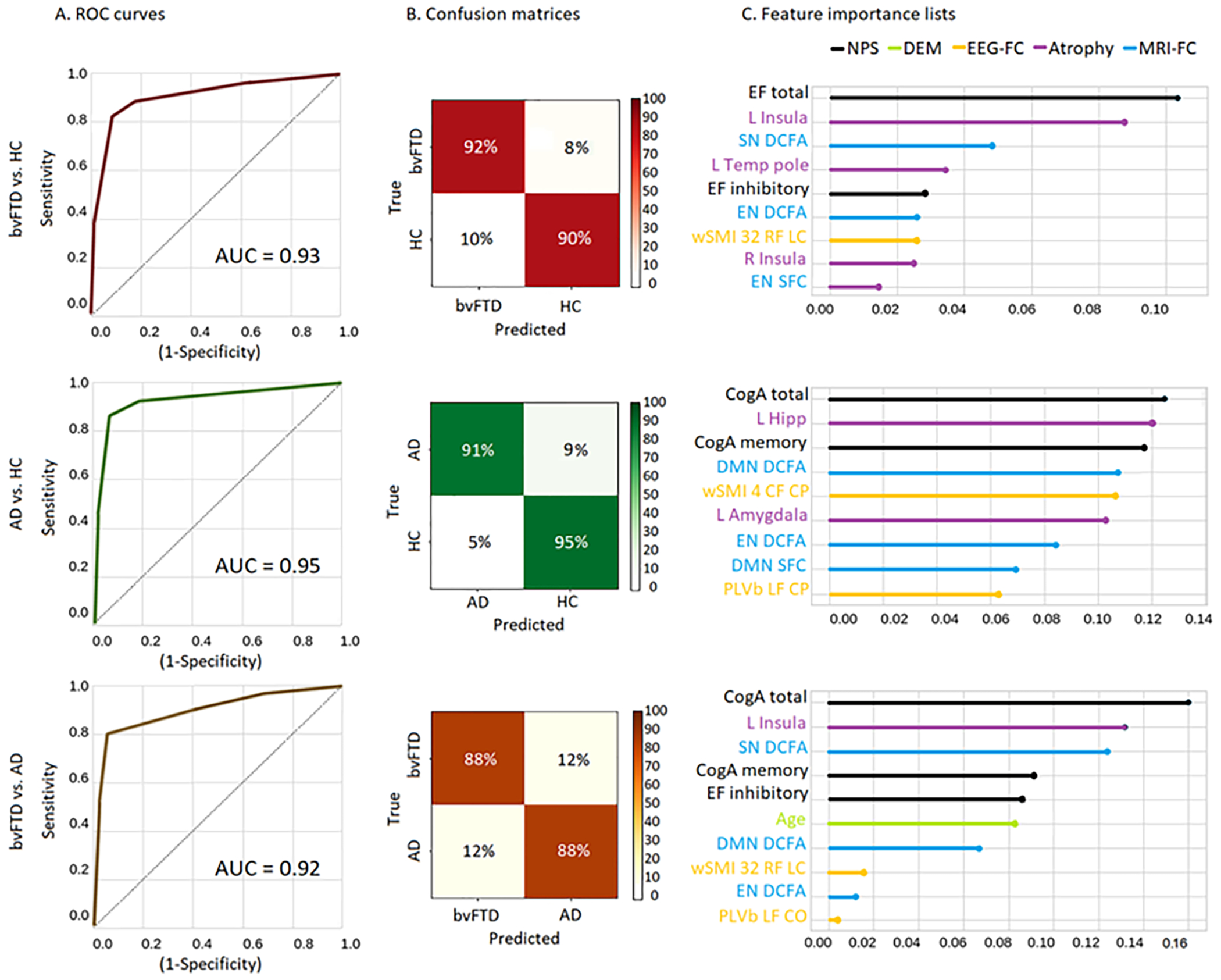
MMAN results for the FS. Machine learning results. (A) BvFTD patients vs HCs. ROC curve indicating specificity (true positive rate) and sensitivity (false positive rate), while calculating the AUC. Confusion matrix for true label and predicted label accuracy details. Feature importance plot of the most relevant features for the classification. Results show an AUC of 0.93, a sensitivity of 92%, and a specificity of 90%, with the EF total value as the top feature, followed L Insula and the SN DCFA as the top-three features. (B) AD patients vs HCs. ROC curve indicating specificity (true positive rate) and sensitivity (false positive rate), while calculating the AUC. Confusion matrix for true label and predicted label accuracy details. Feature importance plot of the most relevant features for the classification. Results yielded an AUC of 0.95, with a sensitivity of 91% and a specificity of 95%. The CogA total value constituted the most important feature, followed by L Hipp and CogA memory as the top-three features (C) BvFTD vs AD patients. ROC curve indicating specificity (true positive rate) and sensitivity (false positive rate), while calculating the AUC. Confusion matrix for true label and predicted label accuracy details. Feature importance plot of the most relevant features for the classification. Results yielded an AUC of 0.92, a sensitivity of 88%, and a specificity of 88%, with the CogA total value as the top feature, followed by L Insula and the SN DCFA as the top-three features. ROC: receiver operating characteristic, AUC: area under the curve; bvFTD: behavioral variant frontotemporal dementia, AD: Alzheimer’s disease; HCs: healthy controls; EF: executive functions; CogA: cognitive assessment; L Insula: left insula; L Temp Pole: left temporal pole; DCFA: dynamic functional connectivity analysis; SN: salience network; DMN: default mode network; EN: executive network; wSMI: weighted symbolic mutual information; PLVb: phase-locking value in the beta band; CF: central-frontal; LT: left-temporal; RF: right-frontal; L amygdala: left-amygdala.

After selecting the optimum features for each classification pair and checking for unbiased results in the FS, we ran the classification analysis between bvFTD patients and HCs using the MMAN with the FS. This classification yielded an AUC of 0.93 (±0.01) in the test set, with a sensitivity of 92% (±3%), and a specificity of 90% (±1%). The feature importance list showed a similar feature profile with respect to the features obtained in the SS classification. The feature importance top-list included the executive function total score as the top feature, followed by left insular atrophy, nonlinear SN, left temporal pole, the inhibition subtest (executive score), the nonlinear EN, right frontal to left central nonlinear EEG connectivity, right insular atrophy, and the linear EN (figure 5, first row). For the classification between AD patients and HCs, we obtained an AUC of 0.95 (±0.01) in the test set, with a sensitivity of 91% (±2%) and a specificity of 95% (±1%). In the feature importance list, the cognitive assessment total score represented the most important feature, followed by left hippocampus atrophy, the memory subtest, nonlinear DMN, nonlinear EEG connectivity, left amygdala atrophy, nonlinear EN, linear DMN, and linear EEG connectivity between the left frontal and central parietal regions (figure 5, second row). Lastly, for the bvFTD vs AD classification, the AUC was of 0.92 (±0.01) in the test set, with a sensitivity of 88% (±1%), and a specificity of 88% (±1%). The feature importance list showed the cognitive assessment (total score) as the top feature, followed by left insular atrophy, nonlinear SN, the memory subtest, inhibition subtest (executive score), age (DEMs), nonlinear DMN, nonlinear EEG connectivity features from right frontal to left central regions, nonlinear EN, and the linear EEG connectivity from left frontal to central occipital regions (figure 5, third row).

### 3.5. Multimodal vs unimodal comparison

**Figure 6.**
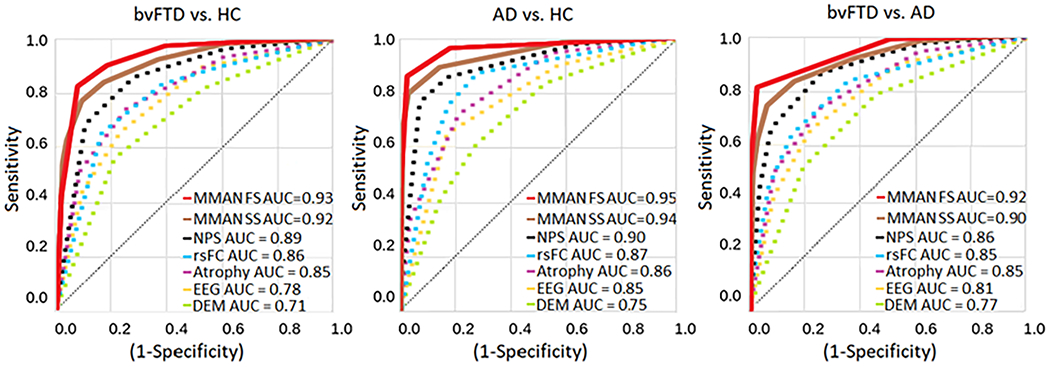
MMAN vs unimodal analysis results. MMAN analysis when using the FS and SS compared to modality-specific results for classification between bvFTD patients and HCs, AD patients and HCs, and bvFTD and. AD patients shown in their respective ROC curves. For the bvFTD vs HC classification, the MMAN FS AUC was 0.93, while the MMAN SS AUC was 0.92, the NPS AUC was of 0.89, for rsFC was 0.86, the Atrophy AUC 0.85, the EEG AUC 0.78, and finally the DEM AUC was of 0.71. For the AD vs HC classification, we obtained an AUC for MMAN FS of 0.95, while the MMAN SS AUC was 0.94, the NPS AUC was of 0.90, the rsFC AUC of 0.87, atrophy AUC was 0.86, the EEG AUC was of 0.85, and lastly the DEM AUC was of 0.75. Lastly, for the bvFTD vs AD classification, the MMAN FS AUC was 0.92, while MMAN SS AUC was 0.90, the NPS AUC was of 0.86, the rsFC AUC of 0.85, the atrophy AUC of 0.85, the EEG AUC of 0.81, and finally the DEM AUC of 0.77. BvFTD: behavioral variant frontotemporal dementia; AD: Alzheimer’s disease; HCs: healthy controls; NPS: neuropsychological markers; MRI-FC: functional connectivity MRI analysis; atrophy: atrophy analysis; EEG-FC: functional connectivity EEG analysis; DEM: demographic values.

To compare our multimodal results (MMAN, both with the SS and the FS) with unimodal analyses, we ran the same preprocessing and machine learning pipeline but using specific feature sets for each modality type (figure 6). To statistically compare the performance between MMAN (SS and the FS) with respect to unimodal approaches, we employed a non-parametric permutation comparison. For all classification pairs, the MMAN yielded higher performance when compared to the individual unimodal approaches. Moreover, the difference in performance was statistically significant in the two MMAN (SS and the FS) with respect to all the unimodal analysis (*p* < 0.05). For the CogA and EF outcomes, we pooled a NPS set. For MRI-FC, we grouped the DCFA and SFC values. All atrophy measures were put together in the atrophy measure. The EEG-FC consisted of the PLV values (comprising all bands) and wSMI (comprising all tau values). Finally, we included DEM variables (sex, age, and years of education). For all classification pairs, the MMAN (both FS and SS) results outperformed unimodal analysis for the three classification pairs (figure 6). For the bvFTD vs HC classification, the MMAN FS AUC was 0.93(±0.01), while the MMAN SS AUC was 0.92(±0.01), the NPS AUC was of 0.89(±0.02), for rsFC was 0.86(±0.03), the Atrophy AUC 0.85(±0.02), the EEG AUC 0.78(±0.04), and finally the DEM AUC was of 0.71(±0.03). For the AD vs HC classification, we obtained an AUC for MMAN FS of 0.95(±0.01), while the MMAN SS AUC was 0.94(±0.01), the NPS AUC was of 0.90(±0.02), the rsFC AUC of 0.87(±0.03), atrophy AUC was 0.86(±0.02), the EEG AUC was of 0.85(±0.03), and lastly the DEM AUC was of 0.75(±0.02). Lastly, for the bvFTD vs AD classification, the MMAN FS AUC was 0.92(±0.01), while MMAN SS AUC was 0.90(±0.01), the NPS AUC was of 0.86(±0.02), the rsFC AUC of 0.85(±0.04), the atrophy AUC of 0.85(±0.03), the EEG AUC of 0.81(±0.02), and finally the DEM AUC of 0.77(±0.04).

## 4. Discussion

The MMAN approach provided support for all the proposed hypotheses on the characterization of AD and bvFTD patients from underrepresented and heterogeneous samples. Using both a SS with complete data and a FS with missing data, the MMAN outperformed all unimodal approaches in classifying bvFTD patients and HCs, AD patients and HCs, and bvFTD and AD patients. MMAN was robust against confounding variables such as multicentric recordings, sociodemographic heterogeneities after harmonization, and overfitting by applying feature reduction techniques. Furthermore, we obtained a modality-specific ranking of classification performance, providing insights on the relevance of different levels of measurements. Overall findings provide a complementary computational framework for diagnosis and characterization of underrepresented populations that can complement dementia assessment in clinical settings.

Our multimodal XGBoost classifier yielded high accuracy and showed similarities with respect to pathophysiological and cognitive profiles registered in unimodal studies on homogeneous populations regarding patterns of atrophy, NPSs, and FC [19, 23, 52, 62]. Top features for the bvFTD vs HC classification were executive dysfunction, insular and temporal atrophy, and non-linear measures of SN connectivity. Executive deficits [10] and insular atrophy [51] are critical in bvFTD. Our feature importance analysis shown that the SN and the ENs were key predictors for this dementia subtype [51]. Moreover, dynamical nonlinearities (DCFA) out-performed statistical and linear methods (SFC) as previously shown [63]. This pattern also emerged when considering EEG connectivity, with non-linear (wSMI) connectivity in frontal hubs emerging as a selected feature that also outperformed the linear measures (PLV). Therefore, our method was able to tap into more complex and comprehensive brain markers of frontal lobe neurodegeneration and nonlinear connectivity.

Similar advantages were found for the classification between AD patients and HCs, with top features involving overall cognitive assessments, hippocampal atrophy, memory-specific cognitive assessments, and non-linear connectivity measures. Overall cognition assessments provided highly accurate AD markers. Direct associations between memory-specific impairments and hippocampal atrophy [64] are observed in this condition in standard neuroradiological assessments for this dementia subtype. Regarding functional neuroimaging, non-linear FC-MRI results mirror previous studies for AD characterization based on the DMN, a network associated with autobiographic memory and specific AD-affected hubs [65], alongside EN alterations that are also present in amnestic mild-cognitive impairment [66]. Moreover, the non-linear FC-EEG measure showed connectivity alterations in fronto-parietal hubs, in line with previous multi-centric study [67] and mirroring broad regions of the FC DMN counterpart [65]. In sum, our results provided a deeper insight into the different pathophysiological markers for this dementia subtype by combining different diagnostic modalities.

Finally, the most clinically relevant prediction was bvFTD vs AD, because such classification requires a more subtle differentiation between neurodegenerative conditions, and not between normal vs neurodegenerative brain health processes. Such accurate differential diagnosis for dementia subtypes is also challenged by the overlapped compromise among conditions [20]. However, cognitive measures (memory and inhibition), insular atrophy, non-linear fMRI and EEG connectivity, as well as age emerged as top features for a high accurate classification. A previous age-matched cohort study comparing overall cognition in bvFTD and AD patients showed distinct patterns of cognitive impairment [68]. In particular, memory impairments and disinhibition are hallmark symptoms of AD and bvFTD, respectively [35]. Further divergent results have been shown in volumetric studies, where specific decreases in gray matter were found in insular regions when comparing FTD to AD [69]. In the MRI-FC connectivity domain, the DCFA on the DMN and SN yielded a high feature importance for differentiating between diseases, in line with previous results on specific network anticorrelations differentiating the two disorders [51]. Moreover, the non-linear EEG connectivity in frontal hubs also confirmed previous reports [70]. Finally, the age-DEM feature appeared as a relevant feature, mirroring differences of disease progression for each dementia subtype [71]. In summary, when assessing data-driven feature differentiation between AD and bvFTD, the model yielded a neurocognitively plausible combination of impairments in specific cognitive domains, together with impairments in specific neural networks differentially affected in each disease. Moreover, results suggest that pathophysiological profiles in neurodegeneration are better described in terms of an integrative approach combining NPS, DEMs, atrophy and non-linear fluctuations of global brain dynamics.

The MMAN provided more accurate dementia characterizations than its unimodal counterparts. When considering modality-specific feature sets for classification, our MMAN (with both the SS and the FS) significantly surpassed NPS, rsFC, Atrophy, EEG, and DEM unimodal classifications. This difference between MMAN (both in FS and SS) and all the unimodal analyses was statistically significant (*p* < 0.05). Possible DEM biases that may have an effect in the multimodal approach were checked in our harmonization analysis showing that the site-specific classification analysis yielded non-significant confusion matrices differences. Moreover, the model performance increase was also statistically significant employing the SS, which had not significant differences in sex, age, and education. Although age appeared as the 4th most important feature in the AD vs FTD classification, this is an expected result since AD onset is usually 10–20 later than FTD onset and can come as a confound when it comes to the detection of sporadic AD [72]. Nevertheless, no other classification had any relevant sociodemographic feature as shown in the feature importance list. Moreover, similar feature profiles were found in FS and SS, pointing those DEM differences had little effect in classification performance. The classification accuracy was subtly improved by adding MRI and EEG to NPS. However, the multifeatured approach was more robust against DEM heterogeneity when compared to NPS tasks alone, which can be biased for specific populations. Moreover, MRI routine diagnostic protocols and EEG affordable markers can be easily incorporated into the dementia assessment to provide a more comprehensive pathophysiological profile.

Our approach successfully integrated cost-effective markers of dementia in a unified computational pipeline that can be implemented in clinical diagnostic setting across developing countries. Other affordable options, such as CSF and plasma biomarkers, are not employed in SACs due to their invasiveness or lack of availability in the region. Major challenges in LMICs, and SACs in particular, involve the lack of expertise available at local centers for the correct interpretation of each diagnostic modality. Similarly, difficulties on grouping a team of experts of each assessment (neuropsychology, MRI/fMRI, EEG) to condense all the interacting factors into a multimodal characterization [73] constitute an important barrier in low-resourced clinical settings. Moreover, multimodal assessments involved routine clinical assessment and methods that are substantially less expensive that PET studies. These, when combined with a robust machine-learning pipeline, constitute a promising approach for centers with limited budgets and infrastructure. Crucially, those protocols should be able to tackle multilevel heterogeneity when employed in variable acquisition contexts [4]. MMAN results similar or better than those of previous PET studies reporting classifications between AD patients and HCs [74] (PET AUC = 0.93 vs MMAN FS AUC = 0.93), bvFTD patients and HCs [75] (PET AUC = 0.89 vs MMAN FS AUC = 0.95), and AD and FTD patients [76] (PET AUC = 0.86 vs MMAN FS AUC = 0.92). These results suggest that, in the absence of PET access, MMAN can provide a complementary option for underrepresented populations. Our approach tackles important clinical tools in the quest for accessible markers in under-represented groups and theoretical implications for a multilevel pathophysiological and neurocognitive characterization of dementia subtypes.

Consortia’s pre-harmonization standards are not massively assessed in UMIC and LMIC. The MMAN was also robust against sources of non-harmonized heterogeneity, such as DEMs (sex, age, years of education), acquisition scanners (1.5 Tesla vs 3 Tesla) and parameters, and missing data. In multicentric data, it is often challenging to balance samples of different DEM backgrounds and acquisition parameters because of population heterogeneity and unequal access to assessments [2, 4, 6, 9]. Moreover, some centers may not have access to specific assessments, resulting in missing data when combining site samples in multi-centric studies. The MMAN provided a harmonization protocol that successfully handled heterogeneity, as reflected in a site-specific confusion matrix from the RFCs analysis. Furthermore, the stability of our results was assessed by using a recursive feature elimination process that allowed us to keep the most stable features (from thousands to dozens), providing optimal classification accuracy and thus preventing overfitting with an adequate combination of multilevel markers. Overall, the reproducibility of our results opens new avenues for optimizing current diagnostic protocols in health centers with variable acquisition settings.

### 4.1. Limitations and future studies

Our work features some limitations. First, AD and bvFTD diagnoses were based on clinical expertise but without pathological or genetic confirmation. However, this limitation is shared by similar works employing traditional statistical and machinelearning techniques to study dementia [19, 51]. Future studies may combine confirmative biomarkers for further assessing the ground truth of patient diagnosis. In this line, our MMAN could also benefit from adding PET imaging, fluid markers, and genetic markers, at least for comparative purposes because of economic constraints in protocol design, to test for synergies between distinct multimodal modalities. Second, the sample size, while limited, was comparable to other multicentric studies of dementia [77]. Thinking forward, more samples from other world regions may be added to test the specificity of the most relevant features in more heterogeneous samples. In the future, we expect to add more ReDLat [4] subject data, with more multimodal features such as genetic, epigenetic, and social determinant of health measures, to test a more detailed profile for dementia characterization. Third, we cannot completely rule out some possible DEM effects in the unmatched sample. In particular, age effects in the AD vs FTD classification are relevant, as current evidence points that age is a critical factor distinguishing both dementia subtype and progression [72]. Future studies may approach age effects in a more systematic way. Finally, these integrative assessments will allow more global comparisons of dementia, by comparing underrepresented samples with those coming from US or Europe.

## 5. Conclusion

In summary, we report a robust pipeline to characterize different measures and deal with regional heterogeneity in underrepresented populations based on low-cost multimodal markers to classify dementia subtypes. These findings highlight the relevance of MMAN for multi-centric studies and clinical settings, where costly biomarkers are unavailable. Moreover, we gained insights into pathophysiological and cognitive profiles for AD and bvFTD, capturing complex associations between clinical, cognitive, atrophy, and nonlinear brain connectivity features. Our approach may improve and facilitate multimodal characterization of dementia that can be used as a complementary decision support tool in clinical settings.

## Figures and Tables

**Table 1.** DEM statistical results for the SS and the FS.

Variable		HCs SS *n* = 54 FS *n* = 152	bvFTD SS *n* = 19 FS *n* = 54	AD SS *n* = 32 FS *n* = 76	Statistics (all groups)	Post-hoc comparisons	
						Groups	*p*-value
Sex (F:M)	SS	26:28	13:6	14:18	*χ*^2^ = 3.14, *p* = 0.21^a^	bvFTD-AD	n.s^b^
						HCs-bvFTD	n.s^b^
						HCs-AD	n.s^b^
	FS	101:51	22:32	24:54	*χ*^2^ = 29.52, *p* < .05^a^	bvFTD-AD	n.s^b^
						HCs-bvFTD	0.001^b^
						HCs-AD	0.001^b^
Age	SS	71.13 (6.12)	68.87 (10.18)	74.02 (5.69)	*F* = 2.85, *p* = 0.07^a^, *ηp*^2^ = 0.06	bvFTD-AD	n.s^c^
						HCs-bvFTD	n.s^c^
						HCs-AD	n.s^c^
	FS	71.54 (7.32)	73.91 (11.63)	76.51 (8.65)	*F* = 2.91, *p* = 0.06^a^, *ηp*^2^ = 0.08	bvFTD-AD	n.s^c^
						HCs-bvFTD	n.s^c^
						HCs-AD	n.s^c^
Years of education	SS	14.16 (3.74)	13.98 (5.19)	12.51 (3.78)	*F* = 2.94, *p* = 0.06^a^, *ηp*^2^ = 0.05	bvFTD-AD	n.s^c^
						HCs-bvFTD	n.s^c^
						HCs-AD	n.s^c^
	FS	15.32 (4.32)	13.76 (5.52)	12.02 (4.41)	*F* = 2.84, *p* = 0.07^a^, *ηp*^2^ = 0.06	bvFTD-AD	n.s^c^
						HCs-bvFTD	n.s^c^
						HCs-AD	n.s^c^

Results are presented as mean (SD). DEM data was assessed through analysis of variance (ANOVAs)—except for sex, which was analyzed via Pearson’s chi-squared (*χ*^2^) test. Effects sizes were calculated through partial eta squared (*ηp*^2^). HCs: healthy controls, bvFTD: behavioral variant of fronto-temporal dementia, AD: Alzheimer’s disease. FS: full-sample. SS: sub-sample.

a *p*-values calculated via independent measures ANOVA.

b *p*-values calculated via chi-squared test (*χ*)^2^.

c *p*-values calculated via Tukey’s range test.

**Table 2.** Missing data distribution in the FS per group.

Feature	HC	bvFTD	AD
CogA abstraction	27%	25%	26%
CogA memory	27%	25%	26%
CogA visuospatial	27%	25%	26%
CogA recognition	27%	25%	26%
CogA attention	27%	25%	26%
EF inhibition	24%	22%	25%
EF conflicting	24%	22%	25%
EF digits	24%	22%	25%
EF proverb	24%	22%	25%
EF motor series	24%	22%	25%
MRI/fMRI	28%	27%	28%
EEG	29%	27%	26%

HCs: healthy controls; bvFTD: behavioral variant of frontotemporal dementia; AD: Alzheimer’s disease; CogA: cognitive assessment; EF: executive functions.

**Table 3.** Specific neuroimaging parameters per center.

	Parameters
Argentina (center 1)	A 3 T Phillips scanner with a standard head coil, whole-brain T1-rapid anatomical 3D gradient echo volumes were acquired parallel to the plane connecting the anterior and posterior commissures, with the following parameters: repetition time (TR) = 8300 ms; echo time (TE) = 3800 ms; flip angle = 8°; 160 slices, matrix dimension = 224 × 224 × 160; voxel size = 1 mm × 1 mm × 1 mm. Also, functional spin echo volumes, parallel to the anterior-posterior commissures, covering the whole brain, were sequentially and ascendingly acquired with the following parameters: TR = 2640 ms; TE = 30 ms; flip angle = 90°; 49 slices, matrix dimension = 80 × 80 × 49; voxel size in plane = 3 mm × 3 mm × 3 mm; slice thickness = 3 mm; sequence duration = 10 min; number of volumes = 220.
Chile (center 2)	Using a 3 T Siemens Skyra scanner with a standard head coil, we acquired whole-brain T1-rapid gradient echo volumes, parallel to the plane connecting the anterior and posterior commissures, with the following parameters: repetition time (TR) = 1700 ms; echo time (TE) = 2000 ms; flip angle = 8°; 208 slices, matrix dimension = 224 × 224 × 208; voxel size = 1 mm × 1 mm × 1 mm. On the other hand, functional EP2D-BOLD pulse sequences, parallel to the anterior-posterior commissures, covering the whole brain, were acquired sequentially intercalating pair-ascending first with the following parameters fMRI parameters: TR = 2660 ms; TE = 30 ms; flip angle = 90°; 46 slices, matrix dimension = 76 × 76 × 46; voxel size in plane = 3 mm × 3 mm × 3 mm; slice thickness = 3 mm; sequence duration = 13.3 min; number of volumes = 300.
Colombia (center 2)	Using a 3 T Siemens Skyra scanner with a standard head coil, we acquired whole-brain T1-rapid gradient echo volumes, parallel to the plane connecting the anterior and posterior commissures, with the following parameters: repetition time (TR) = 2400 ms; echo time (TE) = 2000 ms; flip angle = 8°; 192 slices, matrix dimension = 256 × 256 × 192; voxel size = 1 mm × 1 mm × 1 mm. Finally, functional EP2D-BOLD pulse sequences, parallel to the anterior-posterior commissures, covering the whole brain, were acquired sequentially intercalating pair-ascending first with the following parameters fMRI parameters: TR = 2660 ms; TE = 30 ms; flip angle = 90°; 46 slices, matrix dimension = 76 × 76 × 46; voxel size in plane = 3 mm × 3 mm × 3 mm; slice thickness = 3 mm; sequence duration = 10.5 min; number of volumes = 240.

**Table 4.** Framewise displacement (FD).

	HCs	bvFTD	AD	Stats
Framewise displacement (FD)	0.44 (0.21)	0.51 (0.34)	0.59 (0.31)	*F* = 0.59, *p* = 0.22

FD results are presented as mean (SD). Differences between groups were assessed through ANOVA. Significance was set to alpha level of *p* < 0.05.

HCs: healthy controls, bvFTD: behavioral-variant frontotemporal dementia, AD: Alzheimer’s disease.

## Data Availability

The individual data from this study cannot be shared. Data from the datasets are available for research only after ethical approval for a specific project. The code for the data analysis of this study is available from the corresponding author on reasonable request. The data that support the findings of this study are openly available at the following URL/DOI: https://osf.io/kuvw9/. Data will be available from 29 July 2022.

## Abbreviations

AD: Alzheimer’s disease
bvFTD: Behavioral-variant frontotemporal dementia
HCs: Healthy controls
MRI: Magnetic resonance imaging
fMRI: Functional magnetic resonance imaging
rs-fMRI: Resting-state functional magnetic resonance imaging
EEG: Electroencephalography
LMICs: Low middle-income countries
SACs: South American countries
SES: Socioeconomic status
PET: Positron emission tomography
